# Natriuretic Peptides and Troponins to Predict Cardiovascular Events in Patients Undergoing Major Non-Cardiac Surgery

**DOI:** 10.3390/ijerph19095182

**Published:** 2022-04-24

**Authors:** Marco Alfonso Perrone, Alberto Aimo, Sergio Bernardini, Aldo Clerico

**Affiliations:** 1 Department of Cardiology, University of Rome Tor Vergata, 00133 Rome, Italy; 2 Department of Experimental Medicine and CardioLab, University of Rome Tor Vergata, 00133 Rome, Italy; bernardini@med.uniroma2.it; 3 Fondazione CNR Regione Toscana G. Monasterio, 56124 Pisa, Italy; albertoaimo@libero.it (A.A.); aldoclerico1948@gmail.com (A.C.); 4 Scuola Superiore Sant’Anna, 56124 Pisa, Italy

**Keywords:** cardiac troponins, high-sensitivity methods, cardiac natriuretic peptides, non-cardiac surgery, cardiovascular risk

## Abstract

Patients undergoing major surgery have a substantial risk of cardiovascular events during the perioperative period. Despite the introduction of several risk scores based on medical history, classical risk factors and non-invasive cardiac tests, the possibility of predicting cardiovascular events in patients undergoing non-cardiac surgery remains limited. The cardiac-specific biomarkers, natriuretic peptides (NPs) and cardiac troponins (cTn) have been proposed as additional tools for risk prediction in the perioperative period. This review paper aims to discuss the value of preoperative levels and perioperative changes in cardiac-specific biomarkers to predict adverse outcomes in patients undergoing major non-cardiac surgery. Based on several prospective observational studies and six meta-analyses, some guidelines recommended the measurement of NPs to refine perioperative cardiac risk estimation in patients undergoing non-cardiac surgery. More recently, several studies reported a higher mortality in surgical patients presenting an elevation in high-sensitivity cardiac troponin T and I, especially in elderly patients or those with comorbidities. This evidence should be considered in future international guidelines on the evaluation of perioperative risk in patients undergoing major non-cardiac surgery.

## 1. Introduction

Patients undergoing major surgery have a substantial risk of cardiovascular events during the perioperative period [1,2,3,4,5,6,7]. Although the rate of these events has declined over the past 30 years, they still represent a significant issue in patients undergoing non-cardiac surgery [8,9,10,11], with at least 167,000 cardiac complications of non-cardiac surgical procedures occurring annually in the European Union, 19,000 of which are life-threatening [8]. The 2014 European Society of Cardiology/European Society of Anaesthesiology (ESC/ESA) guidelines recommended that cardiac risk be carefully evaluated in patients undergoing non-cardiac surgery [8]. Despite the development of several risk scores based on medical history, classical cardiovascular risk factors (including sex, age, lipid profile and creatinine concentration), and some non-invasive cardiac tests (such as electrocardiogram, echocardiogram, stress tests), the possibility of predicting cardiovascular events remains limited [4,5,8,9]. This has prompted the assessment of cardiac biomarkers as additional tools for risk prediction [3,10,11,12]. The 2017 Canadian Cardiovascular Society guidelines have recommended the measurement of cardiac natriuretic peptides (NPs) (brain natriuretic peptide, BNP, or N-terminal fragment of proBNP, NT-proBNP) before surgery to refine perioperative cardiac risk estimation [13]. These recommendations were based on prospective observational studies and six meta-analyses evaluating the accuracy of NPs to predict major cardiovascular events after non-cardiac surgery [14,15,16,17,18,19,20,21].

Over the last 10 years, increases in cardiac troponin (cTn) have been associated with an increased short- and long-term risk of cardiac events in patients undergoing different types of non-cardiac surgery [10,11,12,13,22,23,24,25,26,27,28,29,30,31,32,33,34,35,36,37,38,39,40,41,42,43,44,45]. Nonetheless, no specific recommendations on cTn measurement in the perioperative period have been issued so far.

This article aims to discuss the clinical value of preoperative values and perioperative changes in cardiac-specific biomarkers as tools to predict adverse outcomes in patients undergoing non-cardiac surgery.

## 2. Risk Evaluation Using Cardiac Biomarkers in Patients Undergoing Non-Cardiac Surgery

Half of perioperative cardiac deaths occur in patients with no history of heart disease [7,10], suggesting that our current protocols to screen for subclinical heart disease are far from optimal [10]. Risk prediction models based only on clinical criteria, also including classical risk factors and cardiac stress tests, were not shown to improve the accuracy of preoperative risk stratification and to reduce 30-day mortality after non-cardiac surgery [9,11,12]. In particular, a 2019 meta-analysis included six studies on the accuracy of cardiac stress test to predict 30-day mortality [11]. The authors concluded that, despite substantial research, the current body of evidence is insufficient to derive a definitive conclusion as to whether stress testing reduces perioperative mortality [11].

To improve risk stratification and prognostic accuracy in patients undergoing non-cardiac surgery, BNP and NT-proBNP have gained clinical consensus, especially for the detection of subclinical heart failure (HF) [46,47], and cTnI and cTnT for the identification of myocardial damage [43,44,45,46,47,48,49,50,51].

NPs are peptide hormones predominantly produced and secreted mostly by the human heart [43,46,51]. In particular, atrial natriuretic peptides are produced and secreted in the atria, while B-type natriuretic peptides (BNP and the related peptides) are produced and secreted in the ventricles, especially in patients with cardiac disease [43,46,51]. International guidelines recommend the measurement of BNP/NT-proBNP for the diagnosis, risk stratification and follow-up of patients with acute or chronic HF [47,52]. The 2014 ESC/ESA guidelines stated that routine pre-operative NP measurement for risk stratification is not recommended, but may be considered in high-risk patients [8].

More recently, the results of several prospective observational studies and six meta-analyses re-evaluated the prognostic accuracy of NT-proBNP and BNP to predict cardiovascular events after non-cardiac surgery [14,15,16,17,18,19,20,21]. Based on this evidence, the 2017 Canadian Cardiovascular Society Guidelines have strongly recommended BNP or NT-proBNP measurement before surgery to refine perioperative cardiac risk estimation in patients: (1) older than 65 years; (2) aged 45–64 years with cardiovascular disease; (3) with a Revised Cardiac Risk Index (RCRI) score > 1 [13]. The RCRI score includes ischemic heart disease, heart failure (HF), cerebrovascular disease, diabetes mellitus, increased serum creatinine (>177 mmol/L, corresponding to 2.0 mg/dL), and high-risk (major) non-cardiac surgery (defined as intraperitoneal, intrathoracic, or suprainguinal vascular surgery) [13].

The 2014 ESC/ESA Guidelines stated that cTnI and cTnT measurement may be considered in high-risk patients, either before or 48–72 h after major surgery, to detect a myocardial injury [8]. Furthermore, perioperative cTn increases are associated with increased short- and long-term risk of cardiac events in patients undergoing different types of non-cardiac surgery, especially when high-sensitivity (hs) immunoassay methods are used [10,11,12,13,22,23,24,25,26,27,28,29,30,31,32,33,34,35,36,37,38,39,40,41,42].

In 2019, Humble et al. [35] conducted a systematic review and meta-analysis on the prognostic value of increased cTn levels above the cut-off level (usually the 99th percentile upper reference limit (URL)) in adult patients undergoing non-cardiac surgery. Adverse outcome was defined as short-term (in-hospital or <30 days) and long-term (>30 days) major adverse cardiovascular events (MACEs) and/or all-cause mortality non-cardiac [35]. This meta-analysis included 19 studies assessing preoperative cTn and 3 studies evaluating perioperative changes in cTn [35]. These studies assessed preoperative cTn values with a total sample size of 13,386 (range 33 to 4575). They were mainly prospective, single-center studies on patients undergoing a wide range of non-cardiac interventions (mostly intermediate- or high-risk procedures). In particular, hs-cTnT methods were used in just six studies, while the others employed non-hs-cTnI or TnT methods [35]. Preoperative cTn predicted short- (adjusted odds ratio (OR) 5.87, 95% confidence interval (CI) 3.24–10.65, *p* < 0.001) and long-term adverse outcome (adjusted hazard ratio (HR) 2.0, 95% CI 1.4–3.0, *p* < 0.001) [35]. Preoperative cTn predicted short- (adjusted odds ratio (OR) 5.87, 95% confidence interval (CI) 3.24–10.65, *p* < 0.001) and long-term adverse outcome (adjusted hazard ratio (HR) 2.0, 95% CI 1.4–3.0, *p* < 0.001) [35].

More recently, some studies evaluated the utility of perioperative cTn elevation as a prognostic indicator for mortality and cardiac morbidity in patients undergoing surgery for neck of femur fractures [39,53,54]. These fractures are rare in individuals aged <50 years in the absence of high-impact traumas, while in older patients they may be associated with low-velocity trauma because of reduced bone mineral density and frailty [39,53,54]. This meta-analysis included 11 studies with a total of 1363 patients (mean age 83 years, 351 men and 904 women) [39]. Seven studies measured cTnI, three studies measured cTnT and one study used a hs-cTnI assay. Overall, 497 patients (36.5%) experienced a cTn elevation following surgery. Perioperative troponin elevation was significantly associated with all-cause mortality (OR 2.6; 95% CI 1.5—4.6; *p* < 0.001) and cardiac complications (OR 7.4; 95% CI 3.5—15.8; *p* < 0.001) [39]. Increased cTn levels were associated to pre-existing coronary artery disease, cardiac failure, hypertension, previous stroke and previous myocardial infarction [39]. Therefore, perioperative troponin elevation is significantly associated with increased mortality and post-operative cardiac complications in patients operated for neck of femur fractures [39].

## 3. Analytical and Pathophysiological Correlates in Cardiac-Specific Biomarkers

Both NPs and cTn are then useful prognostic indicators in patients undergoing major non-cardiac surgery [10,11,12,13,14,15,16,17,18,19,20,21,22,23,24,25,26,27,28,29,30,31,32,33,34,35,36,37,38,39,40,41,42]. However, clinicians should interpret measured values based on the analytical performance of assay methods and the mechanisms of their production and release from the heart [55,56].

In healthy subjects, NP and cTn are present into the circulation in a range of concentrations (from about 3 to about 50 ng/L) [43,44,45] from 100 to 1000 times lower than other biomarkers, including C-Reactive Protein (CRP), creatinine, cholesterol, D-dimer, Neutral Gelatinase-Associated Lipocalin (NGAL) [43,56,57]. Some circulating proteins and peptides can directly affect the binding of NPs and cTn to specific antibodies of immunoassay methods, interfering with immunoassay methods. Some circulating proteins and peptides can directly affect the binding of NPs and cTn to specific antibodies of immunoassay, interfering with test methods. This interference becomes stronger as the molar concentrations of several substances, able to bind to the specific monoclonal antibodies utilized by immunometric systems, increases compared to that of NP and cTn, as discussed in detail elsewhere [43,44,45,58,59,60]. Furthermore, the measurement of cTnI and cTnT with immunoassay methods can be affected by binding these two cardiac troponins with troponin C, and also with some tissue or plasma proteins, and heterophile- or auto-antibodies to form macro-complexes [58,59]. Therefore, both the accuracy and clinical interpretation of the measured levels of cardiac biomarkers strongly depend on analytical characteristics and performance of assay methods [43,58,59,60,61].

### 3.1. NP Assay

NPs are key diagnostic and prognostic tools in patients with cardiac disease, and are released following every kind of cardiac damage able to activate the neuro-endocrine-immune system. NP increase does not provide any information on the mechanisms of damage acting in the individual patient [51,60,61,62]. In particular, NPs are highly sensitive in detecting cardiac stress in patients with risk factors and/or asymptomatic early vessel damage and/or cardiac dysfunction [51,60,61,62,63].

NPs are rapidly degraded in vivo. The active hormone BNP has a shorter plasma half-life (20–40 min) than the inactive peptide NT-proBNP (>60 min) [51,60,64]. Due to their rapid turnover rate, BNP shows larger intra- (from 40–50%) and inter-individual variability (from 50 to 60%) than those of NT-proBNP (intra 30–40%, inter 40–50%) [60,64]. A large variability means wide confidence intervals and large differences between serial measurements in the same individual, regardless of changes in the disease state [65].

Clinicians should take into consideration some critical issues for a proper interpretation of changes in circulating NPs. Most notably, values should be interpreted according to sex, age, body mass index, comorbidities and therapies [43,47,60,61,62]. In particular, kidney disease can significantly affect NP clearance, increasing BNP and NT-proBNP levels [60,61]. However, a meta-analysis [63] confirmed that NT-proBNP retains utility to diagnose acute HF also in patients with renal dysfunction (although with higher cut-off values) and holds prognostic significance regardless of renal function.

There are larger systematic differences among measured concentrations with the BNP methods than with NT-proBNP methods [60,66]. Due to its greater stability in vivo and in vitro and a smaller difference between assay methods, NT-proBNP is better suited to act as an indicator of disease and prognostic biomarker than BNP [60].

From a clinical perspective, it is important to note that international guidelines recommend cut-off values for the diagnosis and risk prediction in patients with HF, and not for risk stratification in patients undergoing major non-cardiac surgery [46,47]. Cut-off values for evaluation of perioperative risk in patients undergoing major non-cardiac surgery should be defined in specific clinical studies. Optimal cut-off values have been calculated for MACEs. There was wide variation in cut-off values reported in different studies. In 2009, a meta-analysis (including 15 studies with 4856 patients) reported that NT-proBNP cut-off values were higher than those for BNP (range 201–791 ng/L vs. 35–255 ng/L, respectively) [16]. More recently, Rodseth et al. [20] evaluated in an individual patient data meta-analysis the predictive value of preoperative BNP on cardiovascular events (defined as cardiovascular death and nonfatal myocardial infarction) and all-cause mortality during the first 30 days after vascular surgery. Using the receiver operating characteristic (ROC) statistics, the authors calculated the general optimal test cut values for BNP (116 ng/L), as the point that optimizes the rate of true-positive results while minimizing the rate the rate of false-positive results from five data sets [20]. Moreover, the authors proposed several pre-operative BNP cut-off values in predicting 30-day MACEs: 30 ng/L for screening (95% sensitivity, 44% specificity), 116 ng/L for optimal (highest accuracy point; 66% sensitivity, 82% specificity), and 372 ng/L for diagnostic (32% sensitivity, 95% specificity) [20].

### 3.2. cTnI and cTnT Assay

The immunoassay methods with high-analytical sensitivity for cTnI (hs-cTnI) and cTnT (hs-cTnT) are recommended by the most recent international guidelines as gold-standard laboratory methods to detect myocardial injury and diagnose myocardial infarction [49,67,68,69,70]. The document published in 2018 by the American Association for Clinical Chemistry and International Federation of Clinical Chemistry [67] establishes two fundamental criteria that define the hs-cTnI and hs-cTnT methods. The first criterion states that the 99th percentile URL, i.e., the 99th percentile of the distribution of cTnI or cTnT values in the reference population, must be measured with an error (expressed as variation coefficient) of 10% or less. The second, more restrictive, criterion establishes that the hs-cTnI and hs-cTnT methods should measure the biomarker concentrations with values above the limit of sensitivity (limit of detection, LoD) of the assay in at least 50% of individuals of a reference population including at least 300 apparently healthy women and men [67].

The mechanism underlying the presence of detectable troponin levels in healthy subjects is not well understood [48,71,72,73]. Theoretical considerations and some evidence in animals and humans indicate that the 99th percentile URL value corresponds to the amount of cTn present in about 40 mg of myocardial tissue [48,71,72,73]. Cardiac troponins are predominantly located in the cardiomyocyte sarcomere bound to myofibrils, and only about 4–9% of cTnI and cTnT are present in the cytosol of cardiomyocytes as unbound forms [48,71,72,73]. In agreement with these studies, cTn levels in apparently healthy adult subjects should be considered as a specific index, and a close expression of the physiological renewal of cardiomyocytes [48,71,72]. The estimation of the upper reference limit of normality (corresponding to the 99th percentile URL value) of hs-cTnI and hs-cTnT methods is influenced by the demographic characteristics of the reference population, in particular sex and age, and probably also ethnicity [58,59,67,74,75,76]. A very important point is that intra-individual biological variability of these two biomarkers is very low (on average about 9%) [77]. In particular, the hs-cTnI and hs-cTnT methods have a low index of individuality (on average 0.3) associated to a low analytical imprecision (about 5–7% coefficient of variation (CV)) at the 99th percentile URL [58,77]. Due to these favourable indices of variability and imprecision, the critical differences (reference change values, RCV) between two hs-TnI or hs-cTnT values measured in the same individual at different times are lower than other cardiac biomarkers. According to some recent studies, an RCV value >30% can be considered statistically significant for all hs-cTnI and hs-cTnT methods [77,78,79,80,81,82,83]. Therefore, due to the large differences between analytical and biological characteristics between the two cardiac-specific biomarkers, hs-cTnI and hs-cTnT may have a more favourable profile as prognostic biomarkers than BNP and NT-proBNP [58,77,84]. Indeed, the analytical and biological characteristics of hs-cTnI and hs-cTnT methods (summarized in Table 1) are ideal for prognostic biomarkers [44,48,58,60,84].

### 3.3. Combined Measurement of NPs and hs-cTn

According to the 2018 Fourth Universal Definition of Myocardial Infarction [49], the term myocardial injury should be used when there is evidence of elevated hs-cTnI or hs-cTnT concentrations with at least one value above the 99th percentile URL. There are many clinical, cardiac, and extra-cardiac conditions capable of producing myocardial damage without any clinical evidence of acute myocardial ischemia (Table 2). Acute Myocardial Infarction (AMI) is defined as acute myocardial injury with clinical evidence of acute myocardial ischemia with detection of a rise and/or fall of hs-cTnI or hs-cTnT concentrations and at least one value above the 99th percentile URL [49].

Many clinical studies and some meta-analyses have confirmed that there are some individuals apparently free of cardiac disease with hs-cTnI or hs-cTnT concentrations in the third tertile of the distribution values of biomarkers (i.e., still below the 99th percentile URL), who are at higher risk of earlier cardiac or non-cardiac mortality and/or rapid progression to HF [84,85]. The combined measurement of NPs and hs-cTn should detect more easily the individuals at higher risk [44,77]. Indeed, the cardiac-specific biomarkers show different, but complementary, characteristics. Indeed, circulating NPs and cTn may be differently affected by the mechanisms responsible for cardiac dysfunction and/or damage [43,44,56,57,60]. An increment in circulating levels of both biomarkers suggests that stressor mechanisms have already caused relevant alterations on cardiac function (i.e., increased NPs), as well as significant damage to cellular structure (i.e., increased hs-cT [43,44]. A number of experimental and clinical studies reporting that individuals from the general population or patients with cardiac disease show both increased cardiac-specific biomarkers have a more severe outcome than those with only one altered biomarker [84,85,86]. The same notion applies to the setting of non-cardiac surgery. Most notably, Moon et al. [87] retrospectively evaluated 2490 consecutive adult patients undergoing liver transplantation between 2010 and 2018 to determine the prognostic value of BNP and hs-cTnI measurement before liver transplantation to predict post-transplantation mortality. The most important result was that the combined measurement of cardiac-specific biomarkers predicted post-transplantation 90-day mortality: both non-elevated, 1.0%; only one elevated, 9.0%; both elevated, 19.4% (*p* < 0.001; adjusted HR both elevated vs. non-elevated, 4.23 [1.98, 9.03], *p* < 0.001) [87]. Therefore, pre-transplantation BNP and hs-TnI measurement would help define the priority of liver transplantation in individual candidates.

## 4. Evaluation of Myocardial Injury in Patients Undergoing Major Non-Cardiac Surgery

Several studies studied the variations of circulating hs-cTnI and hs-cTnT in patients undergoing major non-cardiac surgery [6,7,8,9,22,23,24,25,26,27,28,29,30,31,32,33,34,35,36,37,38,39,40,41,42,88,89,90,91,92,93].

The VISION trial was a prospective cohort study including 40,004 patients (aged ≥45 years, half of them men) who underwent inpatient non-cardiac surgery at 28 centers in 14 countries, recruited in North and South America, Asia, Europe, Africa and Australia, from 2007 to 2013 [92]. The specific complications evaluated in this study were major bleeding, myocardial injury after noncardiac surgery (MINS), sepsis, non-sepsis infection, acute kidney injury requiring dialysis, stroke, congestive heart failure, venous thromboembolism and new-onset atrial fibrillation. The most common complications were major bleeding (6238 patients, 15.6%), MINS (5191 patients, 13.0%), infection without sepsis (2171 patients, 5.4%) and sepsis (1783 patients, 4.5%). Furthermore, this large multi-center study demonstrated a rate of myocardial injury in non-cardiac surgery (MINS) of 13% (95% CI: 12.7–13.3). MINS was most common in vascular surgery (633 patients, 24.0%) and least common in urologic or gynecologic surgery (503 patients, 10.4%). Moreover, MINS was significantly associated with 30-day mortality (314 deaths; adjusted HR 2.2; 95% CI 1.9–2.6) and was one of the main contributors to death [92]. cTnT was measured at 6–12 h after surgery and on days 1, 2 and 3 after surgery using a non-hs method, which may have led to underestimate the presence of MINS in this trial.

Only the most recent studies used hs-cTnI and hs-cTnT methods to detect MINS [34,37,40,41,88,91]. The most important characteristics of experimental design and clinical results of the studies using hs-cTnI (one study) and hs-cTnT (five studies) are summarized in Table 3. In two retrospective studies, the incidence of MINS was 9% in a study from Spain using hs-cTnI [41], and 3.5% in another study from the USA using hs-cTnT [91]. The other four studies [34,37,40,88] aimed to estimate the association between hs-cTnT and perioperative cardiovascular risk. Despite the large differences in terms of experimental design, the number of patients enrolled, the type of non-cardiac surgery, and outcomes, the mortality rate was clearly higher in patients with MINS compared to those without MINS [34,37,40,88]. Furthermore, patients with MINS more often have other major complications, such as sepsis or bleeding [37,40].

A 2012 study evaluated 46,539 adult patients undergoing non-cardiac surgery in 498 hospitals across 28 European countries [93]. During the follow-up, 1855 patients (4%) died before hospital discharge, 3599 (8%) patients were admitted to critical care after surgery with a median length of stay of 1.2 days (0.9–3.6), and 1358 (73%) patients who died were not admitted to critical care at any stage after surgery [93]. However, crude mortality rates varied widely between countries (from 1.2%; 95% CI: 0.0–3.0 for Iceland to 21.5%; 95% CI:16.9–26.29 for Latvia), possibly because of cultural, demographic, socioeconomic, and political differences between nations affecting health-care outcomes [93].

Overall, the data reported in Table 3 confirm that myocardial injury, evaluated with hs-cTnI and hs-cTnT assay, can be frequently observed in patients undergoing major non-cardiac surgery, especially in older patients or patients with cardiac or extra-cardiac morbidities [34,37,40,41,88,91]. In particular, 11–14% of elevated hs-cTn measurements after surgery were due to a non-ischemic aetiology, while the majority (i.e., 86–89%) were due to myocardial ischaemia [94]. Furthermore, these studies confirm that increased levels of hs-cTnI and hs-cTnT over the 99th percentile URL are significantly associated to an increased risk of mortality or MACEs [34,37,40,88].

## 5. Clinical Considerations about Cardio-Specific Biomarkers Assay in Patients Undergoing Major Non-Cardiac Surgery

There are several technical aspects, perioperative factors, and clinical conditions that can affect the assessment and/or clinical interpretation of cardiac-specific biomarkers in patients undergoing major non-cardiac surgery. Some of these perioperative factors and clinical conditions deserve a more detailed discussion.

### 5.1. Critical Relevance of Biomarker Assay in the Pre-Operative Evaluation

The stress of surgery and anaesthesia may trigger ischaemia by increasing myocardial oxygen demand, reducing myocardial oxygen supply, or both [8]. According to the 2014 ESC/ESA guidelines on non-cardiac surgery [8], the most important aim of a pre-operative evaluation is to check and optimize the control of cardiovascular risk factors in patients undergoing major non-cardiac surgery.

The 2014 ESC/ESA guidelines [8] recommend that NT-proBNP and BNP measurements be considered for independent prognostic information for perioperative and late cardiac events in high-risk patients (class IIb, level B). Furthermore, assessment of cTn in high-risk patients, both before and 48–72 h after major surgery may be considered (Class IIb, Level B). Furthermore, these guidelines suggest that assessment of cTn in high-risk patients, both before and 48–72 h after major surgery may be considered (Class IIb, Level B) [8]. However, the universal pre-operative routine biomarker sampling for risk stratification and to prevent cardiac events is not recommended (Class III, Level C) [8].

We believe that NP and hs-cTn should be measured during pre-operative evaluation [8,23], especially in patients who may be at higher risk, for example, because of their age (>65 years) and comorbidities [84,85]. This proposal is based on pathophysiological and clinical considerations.

The prognostic information related to the assay of NPs and hs-cTn is independent of—and complementary to—other important cardiac indicators of risk, such as ECG and evaluation of cardiac function by imaging techniques, in patients undergoing non-cardiac surgery [6,8,23]. Having elevated levels of cardiac-specific biomarkers before intervention greatly increases the risk of MACEs in the perioperative period [34,35,37,40,87,88].

If a pre-operative measurement is not performed, it is impossible to evaluate the specific contribution of surgery to increased cardiac-specific biomarker levels observed during or soon after intervention.

The hs-cTn assay is able to detect individuals, who are apparently free of cardiac disease, but with increased cardiovascular risk associated to hs-cTn values above the last tertile or even the 99th percentile of the reference population [84,85,95]. It is important to note that there are many clinical, cardiac, and extra-cardiac conditions capable of causing a myocardial injury, especially in elderly patients with co-morbidities (Table 2). Moreover, the presence of elevated cardiac-specific biomarkers in the pre-operative evaluation may suggest to clinicians to perform further laboratory and non-invasive or invasive cardiac testing for the assessment of underlying cardiac disease, and so to obtain additional information on patient clinical condition [2,8].

Several important questions remain to be assessed in dedicated clinical studies, such as how to optimize the management of patients with raised cardiac specific biomarkers before surgery, and whether a strategy based on biomarker measurement improves patient outcomes and the cost–benefit ratio. However, it is conceivable that the relatively low cost due the measurement of hs-cTn (Table 1), performed in the pre-operatory period, should be largely counterbalanced by the benefit of detecting the presence of a myocardial injury in an asymptomatic patient with negative clinical history for cardiac disease, who should undergo to a major non-cardiac surgery intervention without a specific cardioprotective therapy [8,49,75,84,85].

### 5.2. Influence of Cardiac-Protective Drugs

Patients undergoing major non-cardiac surgery with increased cardiac biomarkers, being at risk of MACEs in the peri-operatory period, should be protected from the risk of surgery and anaesthesia stress by using some cardiac-protective drugs [8]. In particular, the 2014 ESC/ESA guidelines [8] recommend that pre-operative initiation of beta-blockers may be considered in patients scheduled for high-risk surgery and who have two clinical risk factors or American Society of Anesthesiologists (ASA) status 3 (Class IIb, Level B). Moreover, beta-blocker therapy before intervention should be considered also in patients with positive clinical history for ischemic heart disease or myocardial ischaemia (Class IIb, Level B) [8]. The primary objective of a cardiac-protective therapy is to reduce the stress on cardiac function by counteracting the activation of neuro-endocrine and immune systems [51,57,62,96]. Accordingly, a continuative therapy with the most popular cardiac-protective drugs (such as beta-blockers, angiotensin-converting enzyme inhibitors, or angiotensin-receptors blockers), tends to reduce the circulating levels of cardiac biomarkers with cardiac disease [51,57,62,96]. Even diuretics, frequently used in patients with hypertension or HF, should be continued in patients undergoing major non-cardiac surgery [8]. Due to the reduction in blood volume, a continuative therapy with diuretics tends to reduce the circulating levels of cardiac-specific biomarkers (especially NP) in patients with hypertension or HF [51,57,62].

### 5.3. Chronic HF

HF is a well-recognized predictor of peri- and post-operative cardiac events and is included in several risk scores [8,97,98,99]. In a study based on a registry analysis of 160,000 Medicare procedures including patients aged ≥65 years, HF was present in 18% and was associated with a 63% increased risk of operative mortality and a 51% greater risk of 30-day all-cause re-admission, compared with patients with coronary artery disease (CAD) or without HF [97]. In particular, one study reported that a reduced left ventricular ejection fraction (LVEF) of ≤35% was found to be a strong predictor of post-operative cardiac events following vascular surgery [99]. According to the international guidelines, circulating levels of cNPs are increased in all patients with chronic or acute HF, both with reduced (HFrEF) or preserved (HFpEF) LVEF [8,47].

Pre-operative levels of NPs (BNP or NT proBNP) are strongly correlated with the outcome of HF and with perioperative and post-operative morbidity and mortality [6,8,18,99]. Furthermore, compared with a pre-operative NP measurement alone, additional post-operative NP measurement improved risk stratification for the composite outcomes of death or non-fatal MI at 30 days and ≥180 days after non-cardiac surgery [20]. Based on this evidence [6,18,20,100], international guidelines [8] recommend that NP assay should be routinely measured in the pre-operative evaluation of patients undergoing major non-cardiac surgery when cardiac dysfunction is known or suspected.

hs-cTnI and hs-cTnT are often elevated in patients with chronic HF, especially those aged > 75 years or with co-morbidities [43,44,45,46,47,50] indicating the presence of HF associated with a non-acute myocardial injury [49]. Increased values of both cardiac-specific biomarkers significantly increase the cardiovascular risk, even in patients undergoing major non-cardiac surgery [1,8,10]. The 2014 ESC/ESA guidelines [8] recommend that specific HF therapy be continued under close monitoring during non-cardiac surgery in stable patients with systolic HF (Class IIa, Level C). Moreover, initiation of specific therapy should be considered at least 1 week before surgery in cardiac-stable patients with systolic HF (Class IIa, Level C) [8]. Importantly, some drugs, such as angiotensin-converting enzyme inhibitors (ACEI) or angiotensin-receptors blockers (ARBs), tend to reduce circulating levels of both cardiac-specific biomarkers [43,44,50,60,96]. In particular, the new combined drug angiotensin receptor-neprilysin inhibitor (ARNI) has complex effects on metabolism of angiotensins and NPs [101,102]. Accordingly, clinicians should consider both the clinical setting and the analytical characteristics of BNP and NT-proBNP methods to correctly interpret variations of NPs measured by commercially available laboratory methods, especially in patients undergoing major non-cardiac surgery and treated with ARNI, in the first month of therapy with this new drug [101,102].

### 5.4. Cardiac Arrhythmias

Cardiac arrhythmias are a frequent cause of morbidity and mortality in the perioperative period [8]. A systematic review (including 14 studies) indicated that increased BNP or NT-proBNP levels can identify patients at risk of postoperative atrial fibrillation (AF), especially after major lung resection or esophagectomy [103].

AF is the most common cardiac arrhythmia. The prevalence of AF increases with age, from less than 0.2% in adults younger than 55 years to about 10% in those 85 years or older, with a higher prevalence in men than in women [104]. Arrhythmias such as AF and ventricular tachycardia often indicate underlying structural heart disease [104]. Atrial fibrillation is a major risk factor for ischemic stroke and is associated with a substantial increase in the risk of stroke [104]. The US Preventive Services Task Force (USPSTF) guidelines published in 2022 state that the current evidence is insufficient to assess the balance of benefits and harms of screening for AF in asymptomatic adults performed by intermittent and continuous screening strategies using ECG technology or photoplethysmography [103]. However, the discovery of such pre-operative arrhythmias should lead to evaluation, including echocardiography, before surgery [8].

Patients with atrial fibrillation usually show elevated NPs (generally more than five times compared with the reference limits in acute episodes) [44]. Due to their rapid turnover in vivo, NP levels tend to decrease rapidly after an acute arhythmic episode (BNP more than NT-proBNP) when patients return to sinus rhythm until values return within normal limits, if a cardiac dysfunction is not present [44,98]. Increased values of hs-cTn are found in about 10–15% of patients with atrial fibrillation and when present they suggest a structural heart disease (i.e., the presence of a myocardial injury) indicating a high cardiac risk.

### 5.5. Acute Myocardial Infarction (AMI)

NPs and hs-cTn above cut-off values before surgery indicate that the patient presents not only a cardiac dysfunction, but also a myocardial injury [44,49,50,60,72,73]. In particular, according to the Fourth Universal Definition of Myocardial Infarction [49], the myocardial injury is present in a patient when a hs-cTn value above the 99th percentile upper reference limit is evaluated in accordance with the laboratory method used for biomarker assay and sex [44,69,70,75]. Furthermore, the term acute myocardial infarction (AMI) should be used when there is acute myocardial injury with detection of a rise and/or fall of cTn values with at least one value above the 99th percentile URL with clinical or instrumental evidence (non-invasive or invasive cardiac imaging techniques) of acute myocardial ischaemia [49]. Indeed, the assay of two or more blood patient samples, collected throughout the perioperative period, are needed to confirm the presence of an acute myocardial injury in a patient with clinical evidence of acute myocardial ischemia [49] (Table 2).

Recent studies confirm that increased hs-cTnI and hs-cTnT values over the 99th percentile URL are significantly associated to an increased risk of mortality or MACEs (Table 3) [34,37,40,88]. The 2014 ESC/ESA guidelines [8] recommend that prophylactic myocardial revascularization before high-risk surgery may be considered, depending on the extent of a stress-induced perfusion defect (Class IIb, Level B). Conversely, these guidelines recommend that routine prophylactic myocardial revascularization before low- and intermediate-risk surgery in patients with proven IHD is not recommended (Class III, Level B).

Even in patients undergoing major non-cardiac surgery, the diagnosis of AMI during or after major non-cardiac surgery should be made by means of hs-cTnI and hs-cTnT assay in accordance with the most recent international guidelines [49,67,68,69,70,105]. In particular, in patients with suspected non-ST-segment elevation MI (NSTEMI), the preference for more rapid algorithms is justified by the consideration that reducing the time for diagnosis promotes an earlier anti-ischemic treatment and then the salvage of larger areas of myocardium still reversibly damaged [68,70,106]. Even if the combined measurement of circulating levels of cardiac-specific biomarkers usually shows better diagnostic and prognostic accuracy and also additive pathophysiological and clinical information in relation to classical invasive and non-invasive cardiac procedures in several clinical conditions (especially heart failure, myocardial injury and acute myocardial infarction), however, clinicians should be aware that both BNP/NT-proBNP and hs-cTn assays may show false (negative or positive) test results.

### 5.6. Alterations in Biomarker Concentration Due to Interfering Substances or Drugs

The analytical performance of immunoassay methods for BNP/NT-proBNP or hs-cTn can be affected by the presence of some interfering substances in the measured sample [58,59,107,108]. The interfering substances of immunoassay methods can be classified in two different types: (1) substances structurally similar to biomarkers, which are able to bind to specific monoclonal antibodies used in the immunometric systems (such as some peptides or drugs); (2) substances able to directly bind the biomarkers, like some plasma proteins or antibodies (such as heterophile antibodies, human anti-animal antibodies, auto-antibodies) [58,59,107,108].

It is important to note that the presence in plasma/serum of an interfering substance can produce false positive, or conversely false negative, results, depending on the immunometric system used (i.e., the interfering effect is strictly method-dependent). Clinicians should suspect an interference when elevated (or reduced) values of a biomarker are constantly measured in plasma/serum samples, collected in different times, using the same immunoassay method without any correlation with the clinical condition of the individual/patient.

It is important to distinguish an immunoassay interference from the pharmacological effect of a drug on the circulating levels of biomarkers. For example, clinicians should take into consideration the effects of some drugs on plasma proteases, inducing an alteration of circulating levels of BNP and NT-proBNP [43,51,60,101,102]. In particular, the drug Entresto (LCZ696) is a fixed-dose combination medication, including a neprylisin (NEP) inhibitor sacubitril and an angiotensin receptor blocker salvartan, which is recommended for the treatment of patients with heart failure [101,102,109]. As the enzyme NEP rapidly degrades the active peptide BNP, but not the inactive peptide NT-proBNP, a significant decrease in NT-proBNP, but not in BNP, is usually observed in the first weeks of therapy with the drug Entresto [101,102]. This fleeting increment (or not decrement) in BNP levels is clearly due to the inhibiting action of sacubitril on NEP enzyme, not to an interference of the drug on the BNP immunoassay system.

On the contrary, the coenzyme biotin (also called vitamin B7) can interfere on some immunoassay systems, which use the streptavidin–biotin interaction for accurately separating the free from bound antigen [110]. Considering that biotin supplementation has progressively expanded over the last years, due to both medically prescribed therapies and vitamin complex preparations purchased for personal dietary supplements, clinicians should take into consideration the assumption of biotin in the case of possible false (negative or positive) results related to BNP/NT-proBNP and hs-cTn immunoassay methods, based on the streptavidin–biotin interaction procedure [110].

From a pathophysiological and clinical point of view, it is important to note that cardiac specific biomarkers (but especially BNP and NTproBNP) are recommended by international guidelines for monitoring the response of patients with heart failure to pharmacological therapy [47,95,96,101,102,109]. Indeed, the rationale for this recommendation is based on the evidence that all the pharmacological treatments able to improve the cardiac function also induce a prompt reduction in circulating levels of cardio-specific biomarkers [47,95,96,101,102]. Conversely, non-responder patients to treatment show no reduction (or, in the more severe cases, a further increase) in circulating levels of biomarkers [47,95,96,101,102].

As recommend by all the international guidelines and expert documents, a close collaboration between clinicians and laboratorians is always needed not only to identify the interfering substances and to choose alternative methods able to measure the cardiac-specific biomarker without any interference, but also to correctly interpret the effects of some drugs on circulating levels of cardiac-specific biomarkers [67,69,75,96,107,108].

## 6. Future Perspectives

Clinical trials are needed to establish whether a management strategy informed by cardiac-specific biomarkers before surgery affects patient outcome. Another important issue is whether the combined measurement of both cardiac-specific biomarkers add significant information to the assay of a single biomarker (NPs or hs-cTn). It is important to note that the assay of hs-cTn may be considered an ideal cardiac biomarker, due to their cardiac-specificity, the relative low cost, and the possibility of obtaining the results with an automated platform within 20–30 min (Table 1) [45,48,50,58,59,60]. Some, very recently commercialized, point-of-care testing (POCT) methods can measure hs-cTnI using only a drop of whole blood with a comparable analytical performance than standard hs-cTn laboratory methods [111,112,113,114,115,116]. Accordingly, these new hs-cTnI POCT assays may allow for a more rapid diagnosis of myocardial injury at the bedside, even in primary care, the intensive care unit or the operating room [111,112,117,118].

## 7. Conclusions

Several recent studies demonstrated the clinical relevance of measuring cardiac-specific biomarkers for cardiovascular risk during the perioperative clinical evaluation of patients undergoing major noncardiac surgery, especially regarding the detection of MINS through hs-cTn methods. However, several questions remain to be addressed in dedicated clinical studies, most notably: (1) how to optimize the management of patients with increased cardiac-specific biomarkers before surgery; (2) if a strategy informed by biomarker measurement can be demonstrated to improve patient outcome and also to have a positive cost/effectiveness profile.

In conclusion, the authors believe that NPs and hs-cTn should also be measured in all patients during the pre-operative clinical evaluation, especially in the case of intermediate or high-risk surgical interventions or in patients aged >65 years and/or at high risk of MACEs because of comorbidities. Of course, in the case that both cardiac-specific biomarkers are increased above the cut-off values (related to method, age and sex), clinicians should accurately evaluate the cardiac function and the possible presence of a myocardial injury in accordance with the international guidelines and expert documents [47,48,68,69,70,75]. Patients with increased cardiac biomarkers, being at high risk of MACEs, should be protected from surgery and anaesthesia stress by using cardiac-protective drugs in accordance with the international guidelines [8].

## Figures and Tables

**Table 1:** Analytical and pathophysiological characteristics of cardiac troponins measured with high-sensitivity methods.

1	Cardiac troponins (especially the cTnI) are produced and released into circulation exclusively by cardiomyocytes, so they are absolutely cardio-specific biomarkers [45,48,50,58,59].
2	Cardiac troponins are more stable in vitro at room temperature than natriuretic peptides [60].
3	Both plasma (with lithium heparin or EDTA) and serum (usually ≤300 mL) can be used for hs-cTnI and hs-TnT assay [58,59,60].
4	Due to their high analytical sensitivity (ranging from 1 to 3 ng/L), hs-cTnI and hs-cTnT methods are able to measure the biomarker levels in the major part of healthy adult subjects [45,48,50,58,59,67].
5	Cardiac troponins have an intra-individual biological variation < 10% CV and an index of individuality of 0.3, i.e., much lower than natriuretic peptides and other cardiovascular biomarkers [64].
6	The laboratory tests for hs-cTnI and hs-cTnT are fully automated and are commercialized at lower cost than other cardiac biomarkers [60].
7	The concentration values can be measured within 30′ min. using the more popular automated platforms for hs-cTn and hs-cTnT methods [45,48,50,58,59].
8	Even if the hs-cTnI and hs-cTnT methods actually show significant differences in measured circulating levels and cut-off values, however, the RCV (Reference Change Value) values are very similar (≥30%), due to their very low intra-individual biological variations and the excellent level of imprecision (about 4–6 CV%) around the cut-off value (i.e., the 99th percentile URL) [58,59,60,65].

**Table 2:** Pathophysiological conditions associated to elevation of measured circulating levels using hs-cTnI and hs-cTnT methods due to the presence of myocardial injury, according to the Fourth Universal Definition of Myocardial Infarction [49].

**Myocardial Injury Related to Acute Myocardial Ischaemia (Related to Type 1 AMI)**
1. Atherosclerotic plaque disruption with thrombosis.
**Myocardial injury related to acute myocardial ischaemia because of oxygen supply/demand imbalance (related to Type 2 AMI)**
1. Reduced myocardial perfusion, e.g.,
Coronary artery spasm, microvascular dysfunction Coronary embolism Coronary artery dissection Sustained bradyarrhythmia Hypotension or shock Respiratory failure Severe anaemia
2. Increased myocardial oxygen demand, e.g.,
Sustained tachyarrhythmia Severe hypertension with or without left ventricular hypertrophy
**Other causes of myocardial injury**
1. Cardiac conditions, e.g.,
Heart failure Myocarditis Cardiomyopathy (any type) Takotsubo syndrome Coronary revascularization procedure Cardiac procedure other than revascularization Catheter ablation Defibrillator shocks Cardiac contusion
2. Systemic conditions, e.g.,
Sepsis, infectious disease Chronic kidney disease Stroke, subarachnoid haemorrhage Pulmonary embolism, pulmonary hypertension Infiltrative diseases, e.g., amyloidosis, sarcoidosis Cardiac procedure other than revascularization Chemotherapeutic agents Critically ill patients Strenuous exercise

**Table 3:** Studies using hs-cTnI and hs-cTnT methods for cardiovascular risk evaluation in patients undergoing major non-cardiac surgery.

Authors (Year)	Method	Type of Study	Enrolled Population	Statistical Results	Ref.
Górka J et al. (2018)	hs-cTnT	Prospective observational cohort study	164 adult patients (≥45 years, men 79.9%, mean age 66.1 ± 9.1 years) undergoing surgery for PAD (88.4%) or AAA (23.8%).	1-year mortality was higher in patients with MINS (23.1%), evaluated by increased hs-cTnT, than non-MINS patients (7.2%; *p* = 0.006).	[34]
Ackland GL et al. (2020)	hs-cTnT	Prospective multicentre observational cohort study	4335 patients aged ≥ 45 years undergoing elective noncardiac surgery (mean age, 65 ± 11 years, men 54.9%).	Patients with elevated troponin (49.8%) have more frequently noncardiac morbidity (OR: 1.95; 95% CI:1.69–2.25), and are also at higher risk of infectious morbidity (OR:1.54; 95% CI: 1.24–1.91) and critical care utilisation (OR:2.05; 95% CI: 1.73–2.43).	[37]
Costa MCDBG et al. (2021)	hs-cTnT	Prospective multicentre observational cohort study	2504 adult (≥45 years) patients (mean age 61.9 ± 11.0 years; men 49%) undergoing noncardiac surgery at two tertiary hospitals.	MINS, evaluated by increased hs-cTnT within 30 days after noncardiac surgery, was related to higher mortality (HR: 3.17, 95% CI: 1.56–6.41), major bleeding (HR 5.76; 95% CI 2.75–12.05), sepsis (HR: 5.08; 95% CI: 2.25–11.46), and active cancer (HR 4.22, 95% CI 1.98–8.98).	[40]
Serrano SK et al. (2021)	hs-cTnI	Prospective cohort with retrospective analysis. Multivariable logistic regression analysis was used to study risk factors associated with MINS, evaluated by increased hs-cTnI levels.	3363 adult (≥45 years) patients (mean age 72.9 ± 11.7 years; men 47.1%) undergoing major non-cardiac surgery.	The incidence of MINS was 9%. Preoperative risk factors that increased the risk of MINS were age, ASA classification and vascular surgery.	[41]
Kler A et al. (2021)	hs-cTnT	Retrospective single centre study	109 consecutive patients (men 48.6%) who underwent open pancreaticoduodenectomy (median age 66 years, range 20–85 years).	ROC curves demonstrated a strong correlation between elevated mean hs-TnT and 30-day (AUC = 0.937), 90-day (AUC = 0.852) mortality and MACEs (AUC = 0.779). In multivariate analysis hs-TnT was significantly associated with 90-day mortality (OR: 43.928, *p* = 0.004) and MACEs (OR: 8.177, *p* = 0.048).	[88]
Turan A et al. (2021)	hs-cTnT	Single centre retrospective analysis	4480 of adults (≥45 years) with routine postoperative TnT monitoring after noncardiac surgery (mean age 62.9 years, men 51.1%).	The incidence of MINS was 155/4480 (3.5%). Lower postoperative haemoglobin values associated with MINS.	[91]

PAD: vascular surgery for peripheral artery disease; AAA: abdominal aortic aneurysm; MINS: myocardial injury after non-cardiac surgery; ASA: American Status Anaesthesiology; MACEs: major adverse cardiovascular events.
